# Frontal Glioblastoma Presenting With Catatonia: A Case Report

**DOI:** 10.7759/cureus.68320

**Published:** 2024-08-31

**Authors:** Shreya S Sinha, Aruna Kaki, Ramya Rachel Jetty, Salma S, Arul Saravanan R

**Affiliations:** 1 Psychiatry, SRM Medical College Hospital and Research Centre, Chennai, IND

**Keywords:** psychomotor disturbances with glioblastoma, catatonic symptoms, glioblastoma tumour, catatonia in glioblastoma, glioblastoma and catatonia, catatonia, frontal lobe glioblastoma, frontal glioblastoma, glioblastoma

## Abstract

This case highlights the need for thorough medical and neurological screening before making any psychiatric diagnosis, even if the patient has a classical syndromic presentation. Presented here is a case of a female in her late 40s coming to our psychiatric outpatient with symptoms suggestive of catatonia. She was treated at a private clinic for depression. As her symptoms deteriorated, she was brought to our hospital for further management. She was diagnosed with catatonia during admission assessment. A detailed neurological assessment later revealed slight weakness in the right upper and lower limb. Following this, a CT scan was done and was reported to have a hypodense area involving the bilateral frontal and basifrontal region, more pronounced on the left side (likely to be acute/subacute). MRI was subsequently done and was found to be suggestive of glioblastoma NOS (not otherwise specified) involving the bilateral cerebral hemisphere which was later confirmed by the histopathology report.

## Introduction

Historically, catatonia as a distinct psychomotor disturbance was first recognized by Karl Ludwig Kahlbaum in 1874 [1] in his book “Catatonia or Tension Insanity” which proved to be a milestone in establishing catatonia as a separate entity but as a part of schizophrenia. However, it is only in the most recent times that our current classificatory systems recognize catatonia as a distinct disorder finding its place as an independent diagnostic entity in the latest version of the International Classification of Diseases (ICD-11) and has also been included in the Diagnostic and Statistical Manual of Mental Disorders, Fifth Edition (DSM-5) [2]. Catatonia comes in three main subtypes: akinetic, characterized by reduced psychomotor activity; hyperkinetic, marked by agitation, combativeness, impulsivity, and purposeless overactivity; and malignant catatonia [3]. It can be manifested in various psychiatric and medical conditions, including catatonia secondary to major depressive disorder, bipolar disorders, schizophrenia, hepatic encephalopathy, stroke, encephalitis, cancers, and others [4].

A brain tumor has two types: primary and metastatic. Primary brain tumors are those that originate from the tissues of the brain or near it and are either glial, originating from the glial cells, or non-glial; they could either be benign or malignant. Glioblastoma multiforme is the most malignant type of glioma, characterized by very active growth, high propensity for invasion, and poor prognosis [5]. It is not uncommon for brain tumors to present with psychiatric symptoms. Catatonia in patients with a brain tumor is a rare presentation [6-8], although psychiatric symptoms, such as depression, are common in cases of brain tumors [9].

This case presents an evolution of psycho-motor symptoms suggestive of catatonia finally being diagnosed as glioblastoma NOS (not otherwise specified) in the frontal region.

## Case presentation

A 48-year-old married female from a rural background, who had studied till primary school, agricultural laborer, hailing from a Tamil-speaking, lower socio-economic, nuclear family, presented to our department with headache, slowness, and inability to perform her daily activities, reduced interaction, poor intake of food, poor self-care for one month, worse over the past 10 days. She also complained of weakness in her right upper and lower limbs and difficulty in walking for the past week. It was insidious in onset and continuous in progression. The patient is a known case of systemic hypertension and is non-compliant with medications. She had no past or family history of psychiatric illness and an adjusted premorbid personality.

The patient was normal one month back when she reported having a headache, dull aching type, not associated with vomiting or blurring of vision. During this period, she isolated herself and had minimal interaction with her family members. She would appear dull and was found to have difficulty in carrying out her day-to-day activities because of her slowness. Her self-care started to deteriorate requiring assistance for the same. As she was withdrawn, dull, and had a poor appetite, the patient was taken to a nearby private psychiatrist where she was prescribed oral psychotropic medications (details of which were not available).

Gradually, the patient’s symptoms worsened and thereby she stopped going to work. She did not do her daily household activities and was found to be lying on her bed most of the day. She would take only liquids such as water, juice, or milk. Her self-care further deteriorated and she lost control over her bowel and bladder. She could no longer communicate verbally. During the same time, she was noticed to have reduced usage of her right hand for her usual chores and was found to drag her right foot while walking, thereby requiring assistance. As her condition was declining, she was brought to our outpatient department for further management.

She entered the room by dragging her right foot and required support from her attendees to sit for the interview. She sat with a drooping posture, leaning to her right side. Eye contact was made but not maintained and her blinking was reduced. Rapport was established with difficulty. The patient was verbally unresponsive to the majority of questions, responding only by nodding her head. She was abnormally still and verbally unresponsive for most part of the interview. At times she would respond to verbal commands. Her effect was dull throughout the interview. On detailed neurological examination, she was found to have sluggish pupil response bilaterally and power of 4/5 on her right upper and lower limb, with flexor plantar response bilaterally. On serial mental status examinations, her effect was dull. Her personal and social judgment was impaired with partial insight. She was uncooperative for further cognitive function tests.

**Figure 1:**
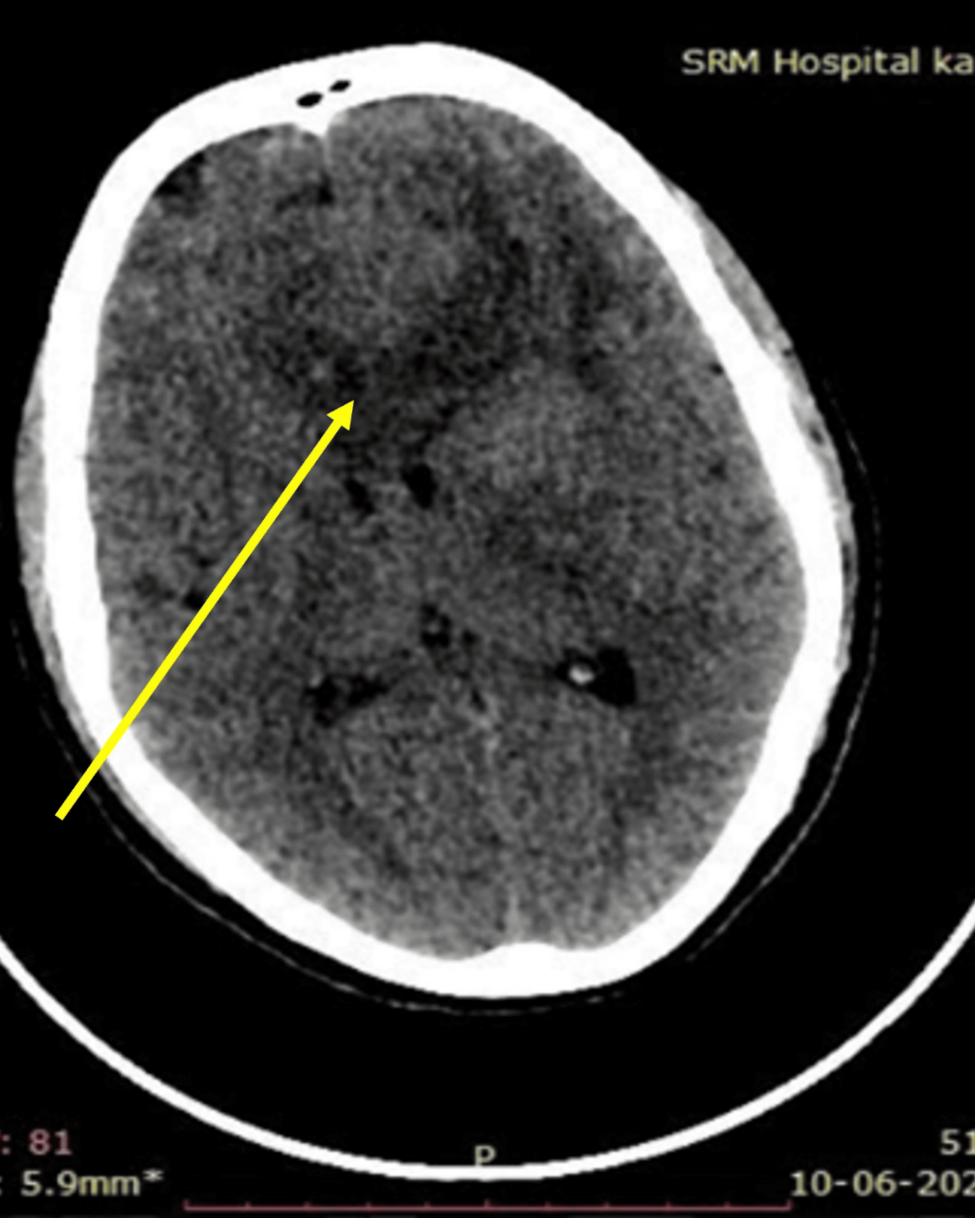
CT brain showing hypodense areas in the bilateral frontal and basifrontal regions, more pronounced on the left side. The hypodense areas extend to the rostrum and body of the corpus callosum on the left side, causing mass effect and midline shift.

**Figure 2:**
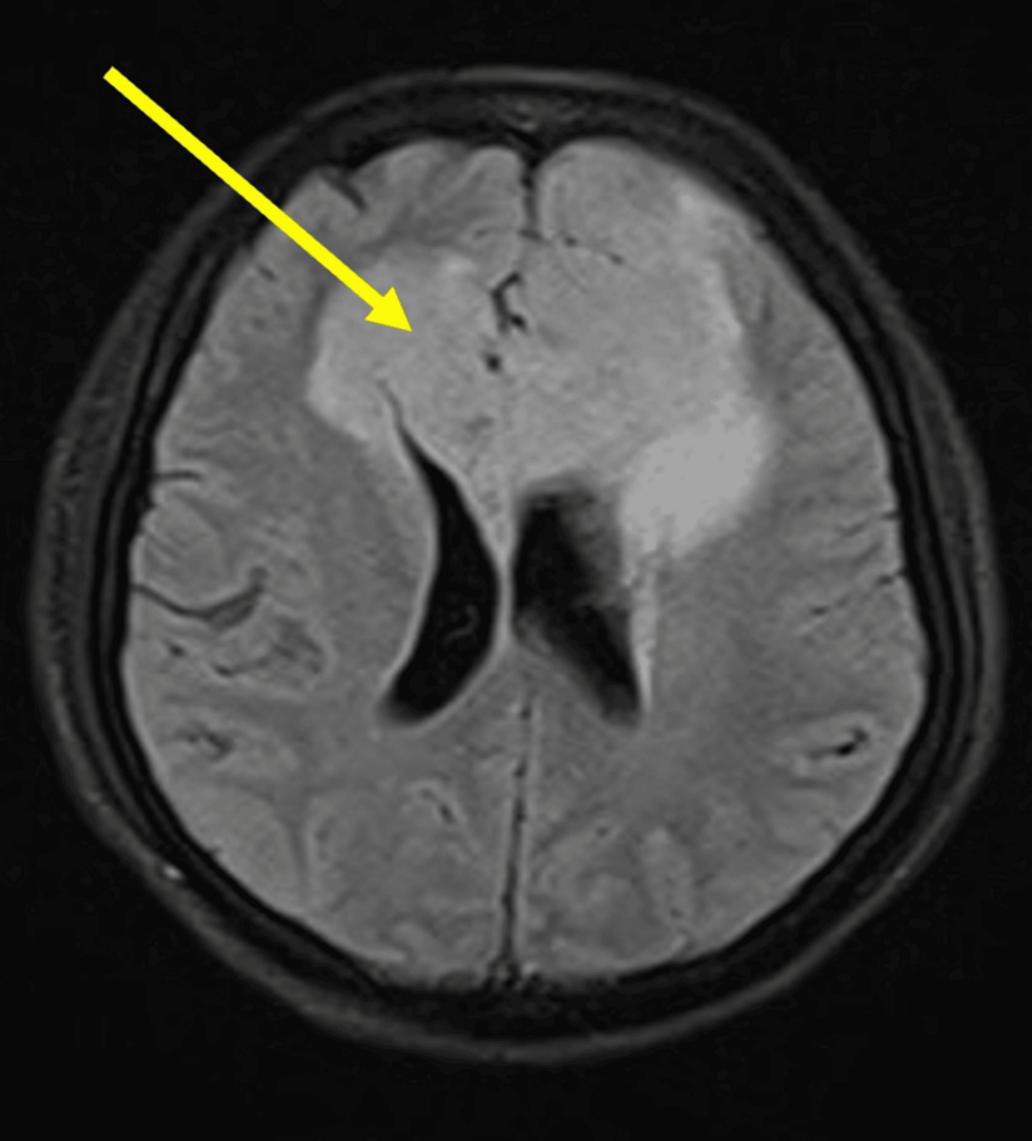
MRI brain showing a large heterogeneous lesion that is hyperintense on T2-weighted and fluid-attenuated inversion recovery (FLAIR) sequences. The lesion is noted in the genu and adjacent body of the corpus callosum, as well as in both frontal lobes and the left basal ganglia.

**Figure 3:**
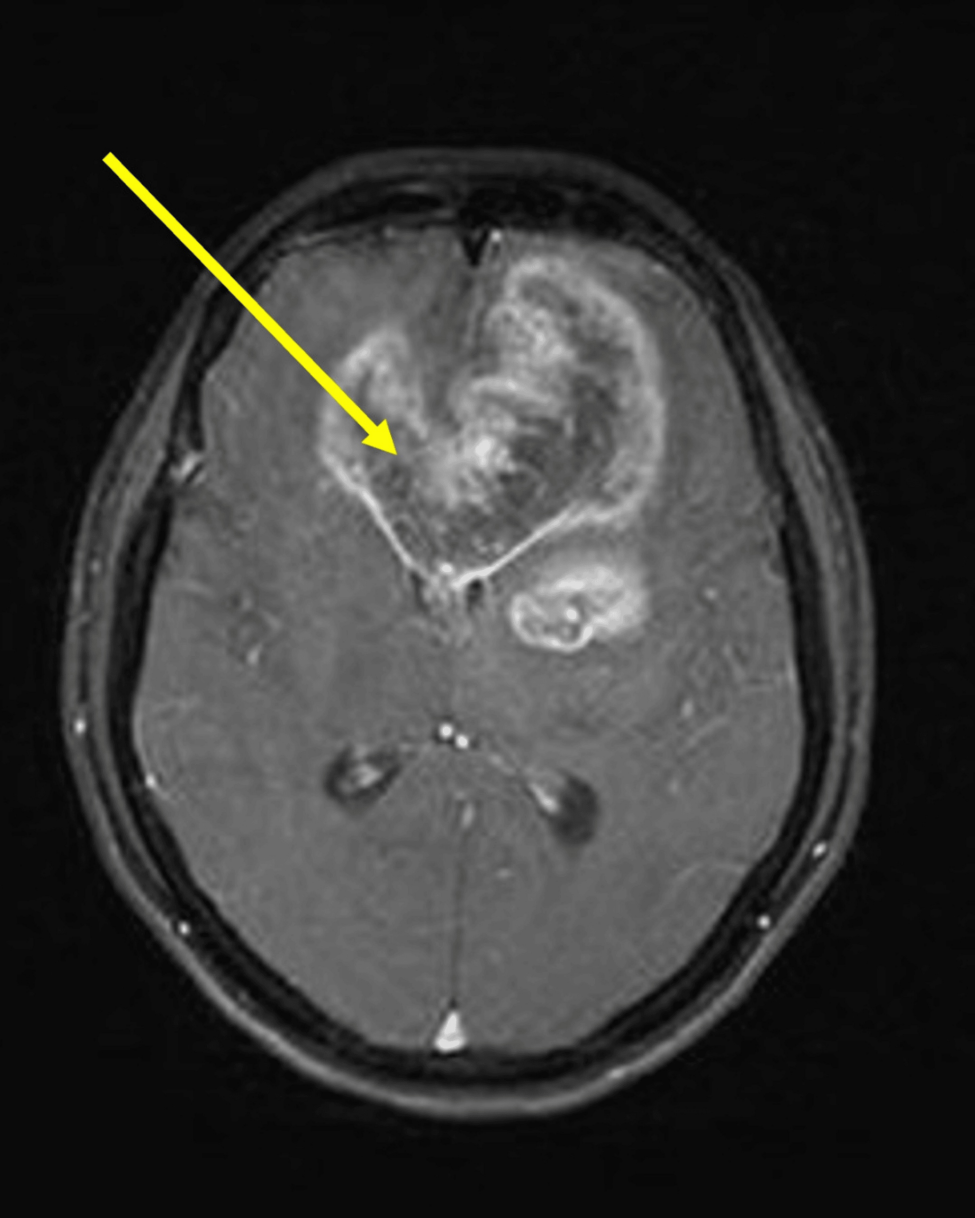
MRI brain showing the lesion with thick, irregular peripheral enhancement on T1 post-contrast imaging. These features are suggestive of glioblastoma.

Her Bush Francis Catatonia Rating Scale (BFCR) scores were 4 in the screening items: immobility/stupor, mutism, and withdrawal. This suggested a positive screening test (i.e. score of more than or equal to 2 out of 14 items) for catatonia. Following this, the whole questionnaire (i.e. from items 1 to 23) was undertaken and maintained a score of 4 on same. Hamilton Depression Rating Scale (HAM-D) scores were 20 suggesting moderate depression. In view of her subacute onset of symptoms, catatonic symptoms, and weakness in her right limbs, CT brain was advised to rule out stroke, hematomas, or any space-occupying lesions that may present similarly as this case. A neurology opinion was sought. CT brain showed a hypodense area involving the bilateral frontal and basifrontal region, more pronounced on the left side extending to the rostrum and body of the corpus callosum on the left side with mass effect and midline shift, also involving the left gangliocapsular region (Figure 1). Therefore, the neurosurgeon recommended an MRI of the brain. The MRI revealed a large heterogeneous lesion that was hyperintense on T2-weighted and fluid-attenuated inversion recovery (FLAIR) sequences, located in the genu and adjacent body of the corpus callosum, as well as in both frontal lobes and the left basal ganglia (Figure 2). On the T1 post-contrast images, the lesion showed irregular peripheral enhancement, which is suggestive of glioblastoma (Figure 3).

**Figure 4:**
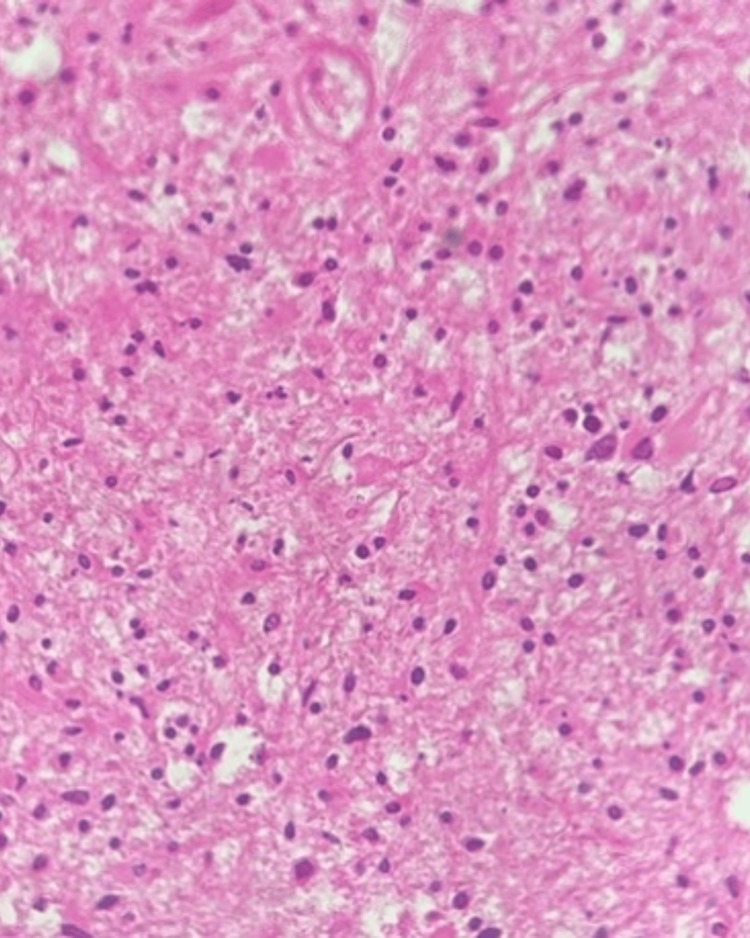
Histopathology showing sections with multiple fragments of an infiltrating cellular tumor of astrocytic origin. The tumor presents as sheets of cells with mild to moderate pleomorphism. The background is fibrillary and contains extensive areas of geographical necrosis and microvascular proliferation, with some vessels displaying a glomeruloid appearance. These features are consistent with a high-grade astrocytic neoplasm, specifically glioblastoma NOS (not otherwise specified).

As she had mild catatonic symptoms scoring in areas of immobility/stupor, mutism, and withdrawal, she was started on an oral dose of lorazepam 2mg BD. Following this, the patient showed minimal improvement in terms of accepting liquids orally and a subtle improvement in depressive or catatonic symptoms. A CT scan was done, a neurology opinion was sought, and the patient was shifted to the Intensive Medical Care Unit, where a neurosurgery opinion was obtained. Prophylactic antiepileptic (levetiracetam) and angioedema (mannitol) measures were advised along with antiplatelets and lipid-lowering agents. In view of the suspicion of a space-occupying lesion upon the CT scan imaging, the neurosurgeon had advised for MRI scan. The patient underwent a right frontal craniotomy and partial excision of the bifrontal tumor. The excised tissue was sent for histopathological examination and it confirmed the diagnosis of glioblastoma NOS (Figure 4).

Post-surgery, the patient’s catatonic and depressive symptoms showed minimal improvement. Her BFCR and HAM-D scores improved from 4 to 3 and 20 to 18 respectively. There was minimal improvement in the patient’s right-sided weakness. The patient was referred to a higher center for further management of the cancerous lesion and was advised to follow up after a month. The overall prognosis of the patient was poor and the same was explained to the patient’s attenders. The patient had lost to follow up and when enquired through telephonic consultation, we learned that the patient expired six months post-surgery.

## Discussion

Catatonia is a syndrome of psychomotor disturbances related to various psychiatric and medical disorders [10]. It is prevalent in 5-18% of psychiatric patients and 3.3% in neurology/neuropsychiatric units [11]. About 20-25% of individuals with catatonic syndrome have an underlying organic cause, with 70% attributed to neurological conditions, primarily structural pathologies of the central nervous system (CNS). Other neurological causes include encephalitis or CNS infections (25%) and seizure disorders (10%).

Brain tumors present with a number of psychiatric co-morbidities of which depression and catatonia are common with depression being the most common presentation [12]. The tumors of the frontal lobe commonly present as depression to psychiatry OPD. In a study by Cheema et al., a 53-year-old male, with a known case of depression for 10 years, had presented with depressed mood, low energy, anhedonia, and reduced sleep. Given his poor response to antidepressants and history of seizures, a CT scan followed by an MRI was done. The imaging revealed a mass in the left frontal and temporal lobes extending into the right frontal lobe. Subsequent brain biopsy confirmed the presence of glioblastoma multiforme [13].

Tumors in the brain's frontal lobe, especially in the prefrontal cortex (PFC), can cause a psychopathological condition affecting behavior, personality, memory, and social interactions [14]. In our case, the patient initially presented with symptoms suggestive of depression which later progressed to develop psychomotor disturbances suggestive of catatonia. Eventually, the imaging revealed the underlying glioblastoma involving bilateral frontal lobes with extension in the left basal ganglia and corpus callosum. The histopathology report further confirmed the diagnosis.

This finding is similar to that of a case report by Arora and Praharaj, wherein a patient with catatonia was later found to have butterfly glioma originating from the corpus callosum with bilateral medial frontal extensions. A 45-year-old male presented with a three-month duration of increased frequency of micturition and a month’s history of forgetfulness, crying spells, decreased socialization and hallucinatory behavior with food refusal, maintaining odd postures, and urinary incontinence for one week. On mental status examination, the patient was uncooperative, mute, and had posturing and negativism. The patient was given lorazepam 2mg injection TID; however, no improvement was observed. The CT brain then revealed a large butterfly glioma involving the corpus callosum's genu and body extending into the medial frontal lobe. The patient eventually succumbed to death [7].

The above study confirmed that medial frontal lobe structures have been implicated in the pathophysiology of catatonia. Taylor noted that the presence of catatonic symptoms is a piece of evidence for frontal lobe disease or dysfunction, and has proposed it to be due to dopamine imbalance in the frontal lobe, basal ganglia, and brain stem system [15].

Other than frontal lobe lesions, psychiatric symptoms of depression and catatonia have also been seen in temporal lobe lesions. In the study by Franssen and Sienaert, a 57-year-old man, initially presented with symptoms of depression. His referral to the hospital was prompted by a sudden worsening of his depressive state. Upon evaluation, he was diagnosed with catatonia and effectively treated with lorazepam. An intracranial mass accompanied by uncal herniation and mass effect was discovered in the MRI scan and was later confirmed as glioblastoma multiforme on the histopathology report. Numerous cases have documented instances of catatonia occurring in temporal abnormalities, which include temporal lobe infarction, localized temporal encephalomalacia, surgical resection, or lesions of the temporal lobe [16].

A recent critical literature review also highlighted that patients with catatonia require detailed medical evaluation for CNS-related causes [17]. Catatonia is associated with significant morbidity and mortality if left untreated. With the early diagnosis and management of the underlying condition, catatonic symptoms resolve and it further improves the prognosis. Thereby necessitating the need for proper history and physical and neurological examination in the patients having catatonic symptoms.

## Conclusions

Any new onset of behavioral changes warrants a thorough screening for medical and neurological causes. Any acute onset psycho-motor symptoms like headache or catatonia must be evaluated with a complete neurological assessment, supplanted with neuroimaging studies, like CT or MRI of the brain, before making a psychiatric diagnosis. The appearance of catatonic symptoms should prompt clinicians to rule out organic causes, especially neurological causes. Early diagnosis and intervention can significantly impact the outcome and prognosis of the underlying medical condition.
